# Parkinson’s disease-associated, sex-specific changes in DNA methylation at *PARK7* (DJ-1), *SLC17A6* (VGLUT2), *PTPRN2* (IA-2β), and *NR4A2* (NURR1) in cortical neurons

**DOI:** 10.1038/s41531-022-00355-2

**Published:** 2022-09-23

**Authors:** Joseph Kochmanski, Nathan C. Kuhn, Alison I. Bernstein

**Affiliations:** Department of Translational Neuroscience, College of Human Medicine, Michigan State University, Grand Rapids, MI USA

**Keywords:** Parkinson's disease, Parkinson's disease, Epigenetics analysis

## Abstract

Evidence for epigenetic regulation playing a role in Parkinson’s disease (PD) is growing, particularly for DNA methylation. Approximately 90% of PD cases are due to a complex interaction between age, genes, and environmental factors, and epigenetic marks are thought to mediate the relationship between aging, genetics, the environment, and disease risk. To date, there are a small number of published genome-wide studies of DNA methylation in PD, but none accounted for cell type or sex in their analyses. Given the heterogeneity of bulk brain tissue samples and known sex differences in PD risk, progression, and severity, these are critical variables to account for. In this genome-wide analysis of DNA methylation in an enriched neuronal population from PD postmortem parietal cortex, we report sex-specific PD-associated methylation changes in *PARK7* (DJ-1), *SLC17A6* (VGLUT2), *PTPRN2* (IA-2β), *NR4A2* (NURR1), and other genes involved in developmental pathways, neurotransmitter packaging and release, and axon and neuron projection guidance.

## Introduction

Parkinson’s disease (PD) is the second most common neurodegenerative disorder in the US, and is characterized by progressive degeneration of dopaminergic neurons in the nigrostriatal pathway and the formation of α-synuclein-containing Lewy bodies^1^. An estimated 5–10% of PD cases are familial and several genes have been identified that cause these inherited forms of the disease^2,3^. The remaining ~90% of cases are likely due to a complex interaction between age, genes, and environmental factors. Given that epigenetic marks are dynamic with age, sensitive to the environment, and regulate gene expression throughout the lifespan, they are considered a potential mediator of the complex relationship between aging, genetics, the environment, and disease disease^4,5^.

Evidence for the role of epigenetic regulation in PD is growing, particularly for DNA methylation^6–12^. In particular, previous studies have reported differential DNA methylation at PD-related genes (*MAPT*, *CYP2E1*, and *STX1B*)^13–15^, and hypomethylation of the α-syn gene (SNCA) is associated with decreased protein levels in the substantia nigra (SN) and striatum of postmortem PD brain^16–23^. In addition to these gene-specific studies, genome-wide analyses of DNA methylation from postmortem PD brain tissue have identified a number of gene regions that show differential DNA methylation in PD brains^24–27^. Finally, a case-control study identified an association between a polymorphism in PD risk and DNA methyltransferase 3B (DNMT3B), which is responsible for establishing de novo patterns of DNA methylation during embryonic development^28^. Taken together, these multiple lines of evidence support the role for dysregulation of DNA methylation in the etiology of PD.

However, the existing genome-wide analyses of DNA methylation in PD have not adequately addressed the effects of sex or cell-type heterogeneity. Given that men and women show differences in PD risk, disease progression, and disease severity^29–31^, it is critical to include sex in experiments examining the potential role for the epigenome in PD. Furthermore, the majority of existing studies of epigenetics in PD have utilized either blood or bulk brain tissue samples^24,26,32,33^. Although DNA methylation changes in blood are useful for identifying biomarkers, they may not be representative of a pathogenic mechanism in the brain. Further, analyses from bulk brain tissue cannot address the cell-type specificity of identified changes, making it difficult to determine which cell types may be driving PD-related differential DNA methylation. In a step forward for the field, one recent paper investigated DNA methylation in an enriched neuronal population using fluorescence-activated cell sorting, but only explored DNA methylation at enhancer regions, not genome-wide^27^. Here, we report the results from a genome-wide analysis of DNA methylation in enriched neurons from PD brain stratified by sex.

In this study, we obtained human postmortem parietal cortex (*n* = 50 control, *n* = 50 Parkinson’s disease) from the Banner Sun Health Research Institute Brain Bank and enriched for neuronal populations using magnetic-activated cell sorting (MACS) for the neuronal marker, NeuN. Postmortem parietal cortex was used because this region develops pathology in the late stages of PD and is expected to still have robust populations of neurons, from which disease-affected gene regulatory marks can be measured. Genome-wide DNA methylation was measured using the Illumina EPIC BeadChip array paired with bisulfite treatment. Bisulfite treatment is a well-established method for measuring DNA methylation, but it actually cannot distinguish between DNA methylation and DNA hydroxymethylation^34,35^. As such, our dataset actually measures both DNA methylation and DNA hydroxymethylation without discriminating between these two epigenetic marks. Despite this important caveat, for simplicity’s sake and to match prior literature, we will discuss our results as changes in “DNA methylation.”

We also tested the hypothesis that PD is associated with accelerated epigenetic aging in the brain. Aging is the primary risk factor for PD, and previous work has shown that aging and PD progress via shared cellular mechanisms^36–39^. Multiple hypotheses have been proposed to explain the association between aging and the development of PD, including the ‘multiple hit’ hypothesis and the ‘stochastic acceleration hypothesis’^37,40^. In general, these hypotheses work under a similar biological model – that an accumulation of factors, both genetic and environmental, accelerate the normal pace of dopaminergic neuron loss with age, eventually exceeding a threshold for PD diagnosis^37^. However, the biological mechanism by which environment and genetics interact to accelerate age-related dopaminergic neuron loss remains unclear. Because the epigenome is dynamic with age and shows programmed age-related changes, it has been proposed that acceleration of these age-related changes can contribute to disease risk in the aged human^41–44^. Supporting this idea, studies have shown associations between accelerated epigenetic aging and a variety of disease states, including a study using blood samples from PD patients^32,45^. However, to our knowledge, no existing studies have investigated whether neurons show altered epigenetic aging in PD brains compared to control.

## Results

### Human brain tissue sample selection

De-identified tissue samples from control (*n* = 50) and Parkinson’s disease (*n* = 50) human brain samples were obtained from archival human autopsy specimens provided by the Banner Sun Health Research Institute (BSHRI), using BSHRI’s approved institutional review board (IRB) protocols. Further details about the BSHRI’s brain samples are available in a previous publication^46^. We selected PD patients with mid-stage disease (Braak stage = II–III), as defined by Lewy pathology^47^. The cohort of control brains consisted of patients who died from non-neurologic causes and whose brains had no significant neurodegenerative disease pathology. Subjects were split by sex (*n* = 63 males, *n* = 37 females), and data for the sample cohort are summarized in Table 1. For each subject (*N* = 100), the parietal cortex was obtained. This region develops pathology late in PD; in mid-stage PD, the parietal cortex is expected to still have robust populations of neurons (unlike the substantia nigra, where neuron loss occurs early in disease), providing an avenue to investigate pre-pathological changes in gene regulation.

**Table 1:** Cohort characteristics including disease status, age at death in years, postmortem interval (PMI) in hours, and race.

	Male (*N* = 63)		Female (*N* = 37)	
Variables	Mean ± SD or *N* (%)	Range	Mean ± SD or *N* (%)	Range
*Disease status*				
Control	30 (47.6%)		20 (54.1%)	
Parkinson’s disease	33 (52.4%)		17 (45.9%)	
*Age at death*				
Control	79.1 ± 9.1	53–93	82.2 ± 13.1	52–95
Parkinson’s disease	79.2 ± 7.4	64–95	79.4 ± 5.5	70–87
*Postmortem interval (PMI)*				
Control	3.28 ± 0.82	2.16–5.5	3.05 ± 0.97	1.25–5
Parkinson’s disease	3.28 ± 0.86	1.83–5.23	3.19 ± 0.8	1.75–4.42
*Race*				
White	62 (98.4%)		37 (100%)	
Unidentified	1 (1.6%)		0 (0%)	

One male was missing race data. One male control sample was removed during quality control, leaving *n* = 99 in the final analyses.

*SD* standard deviation.

### Validation of neuronal enrichment

**Fig. 1:**
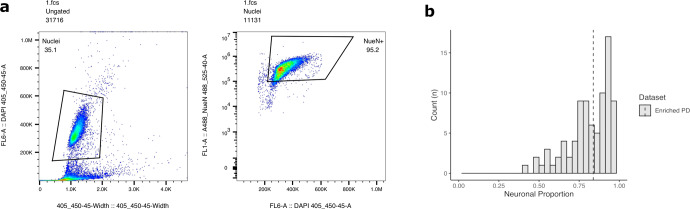
Neuronal enrichment validation by flow cytometry and CETS analysis. **a** NeuN^+^ nuclei were identified using DAPI pulse area vs. width using the 405-450/45 channel for all nuclei (left) followed by NeuN-AlexaFluor488 bright events 488-525/40 (right). The percentage of NeuN^+^ nuclei is provided in the top-right corner. **b** Histogram of estimated neuronal proportion (vs. glial) for enriched PD NeuN^+^ nuclei population. Proportions of neuronal and glial cell types were estimated for MACS-sorted NeuN^**+**^ nuclei samples using the *CETS* R package during EPIC array data processing. Average proportion of neurons (vertical dotted line) was estimated to be 83.8% across all *n* = 99 samples included in analysis.

To validate the enrichment of NeuN^+^ nuclei via MACS prior to experimentation, we quantified total nuclei and NeuN^+^ nuclei for six validation samples using flow cytometry. A representative quantification plot is included in Fig. 1a. Plots for all six validation samples are available as a supplementary file (Supplementary Fig. 1). For the validation samples, the mean % NeuN^+^ was 92.0% (range = 89.2–95.2%). As a secondary validation, we estimated proportions of neuronal and glial cell types for all experimental MACS-sorted parietal cortex samples using the Cell Epigenotype Specific (*CETS)* package during EPIC array data processing. One sample from the male control group that was not one of the six with validation by flow cytometry had an estimated neuronal population of only 0.03% in the *CETS* analysis. This sample was removed prior to all downstream differential methylation testing. The average proportion of neurons in the remaining 99 samples was estimated to be 83.8% by *CETS* analysis (Fig. 1b).

### Differential testing for differentially methylated CpGs

In our sex-stratified models for differential DNA methylation testing, we identified 3 differentially methylated CpGs (DMCs) in males and 87 significant DMCs in the female parietal cortex associated with PD (*p*-value < 9 × 10^−8^) (Additional files 1 and 2). This *p*-value cutoff was selected based on recommendations in a recent study of epigenome-wide association studies^48^. The sex-stratified DMC models included a sigma term to test for differences in the mean by disease state while accounting for potential differences in variability. The male DMCs annotated to 3 unique gene IDs and the female DMCs annotated to 85 unique gene IDs; two female DMCs annotated to the same gene. These results were sex-specific, and there was no overlap between any of the identified male and female DMCs by probe ID.

**Fig. 2:**
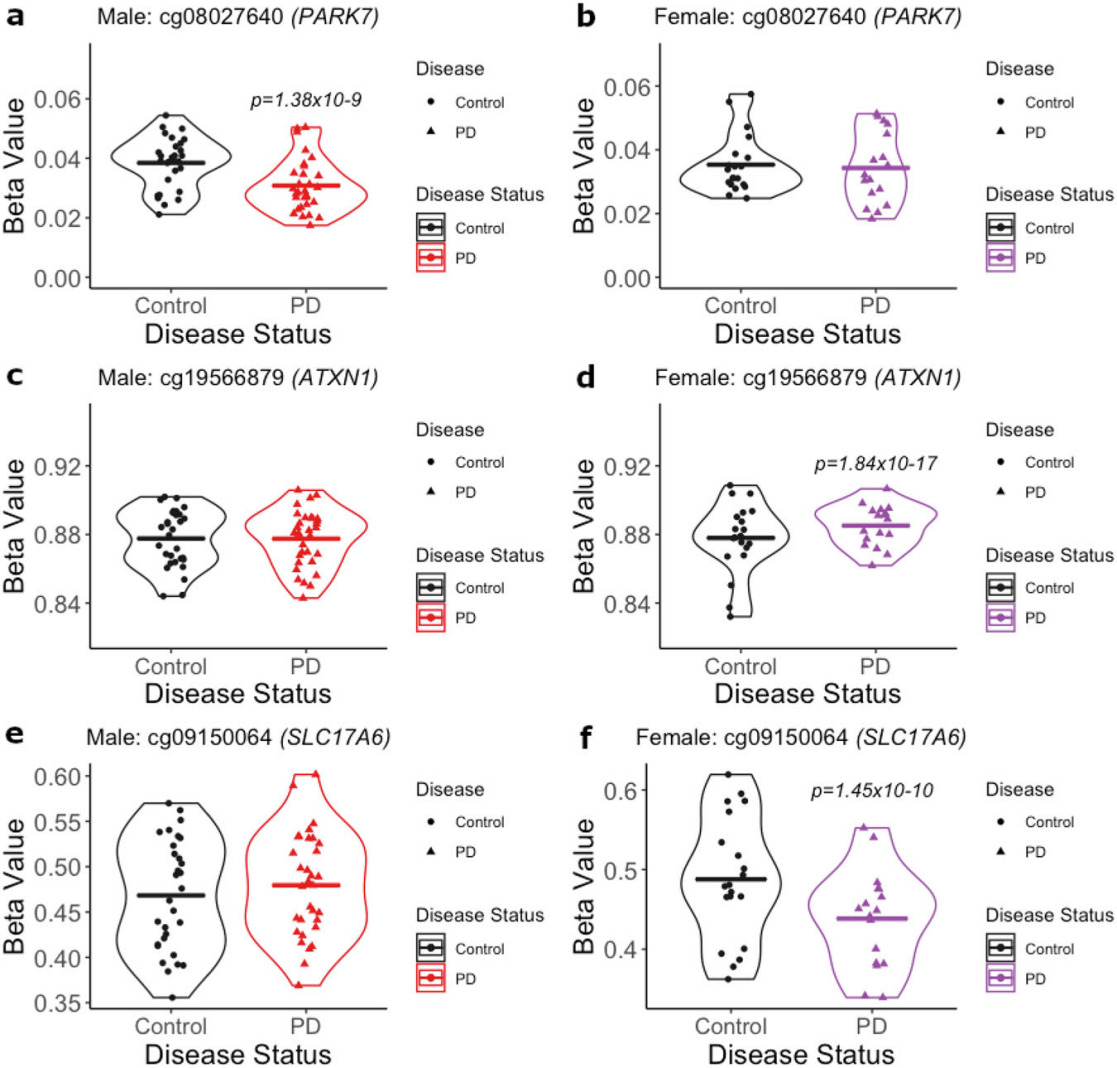
Dot plots of significant male and female DMCs by Parkinson’s disease. **a** In males, the most significant DMC was annotated to the *PARK7* locus (cg08027640; *p*-value = 1.38 × 10^−9^). **b** There was no significant change in DNA methylation at cg08027640 in females. **c** There was no significant change in DNA methylation at cg19566879 in males. **d** In females, the most significant DMC was annotated to the *ATXN1* locus (cg19566879; *p*-value = 1.84 × 10^−17^). **e** There was no significant change in DNA methylation at cg09150064 in males. **f** In females, a significant DMC was annotated to the *SLC17A6* locus (cg09150064; *p*-value = 1.45 × 10^−10^). *P*-values indicate significant change in PD compared to control (cutoff for significance: *p*-value < 9 × 10^−8^).

Here, we highlight the most significant DMCs and a DMC annotated to a gene of interest for PD. The most significant DMC in males (*p*-value = 1.38 × 10^−9^) was located within the *PARK7* locus and showed male-specific hypomethylation in PD cortical neurons compared to control (Fig. 2a, b). Visualization of the identified DMCs in the Wash U Epigenome Browser with NIH ROADMAP Epigenomics chromatin state predictions show that this DMC is located in a CpG island flanking the transcription start site (Fig. 4a)^49,50^. In females, the most significant DMC was located within the gene body of the *ATXN1* gene (*p*-value = 1.84 × 10^−17^). This *ATXN1* DMC is located within a predicted enhancer associated with weak transcriptional activity and showed female-specific hypermethylation in PD cortical neurons compared to control (Figs. 2b, 4d)^49,50^. We also identified a female-specific hypomethylated DMC within the gene body of the *SLC17A6* locus (*p*-value = 1.45 × 10^−10^) (Figs. 2c, d, 4b)^49,50^.

### Differential testing for differentially methylated regions

In our sex-stratified models for differential genomic region testing, we identified 258 significant differentially methylated regions (DMRs) in males and 214 significant DMRs in female parietal cortex by Parkinson’s disease (minimum smoothed FDR < 0.05) (Additional files 3 and 4). These DMRs annotated to 205 unique gene IDs in the males and 157 unique gene IDs in the females. Here, we highlight 3 DMRs of interest.

**Fig. 3:**
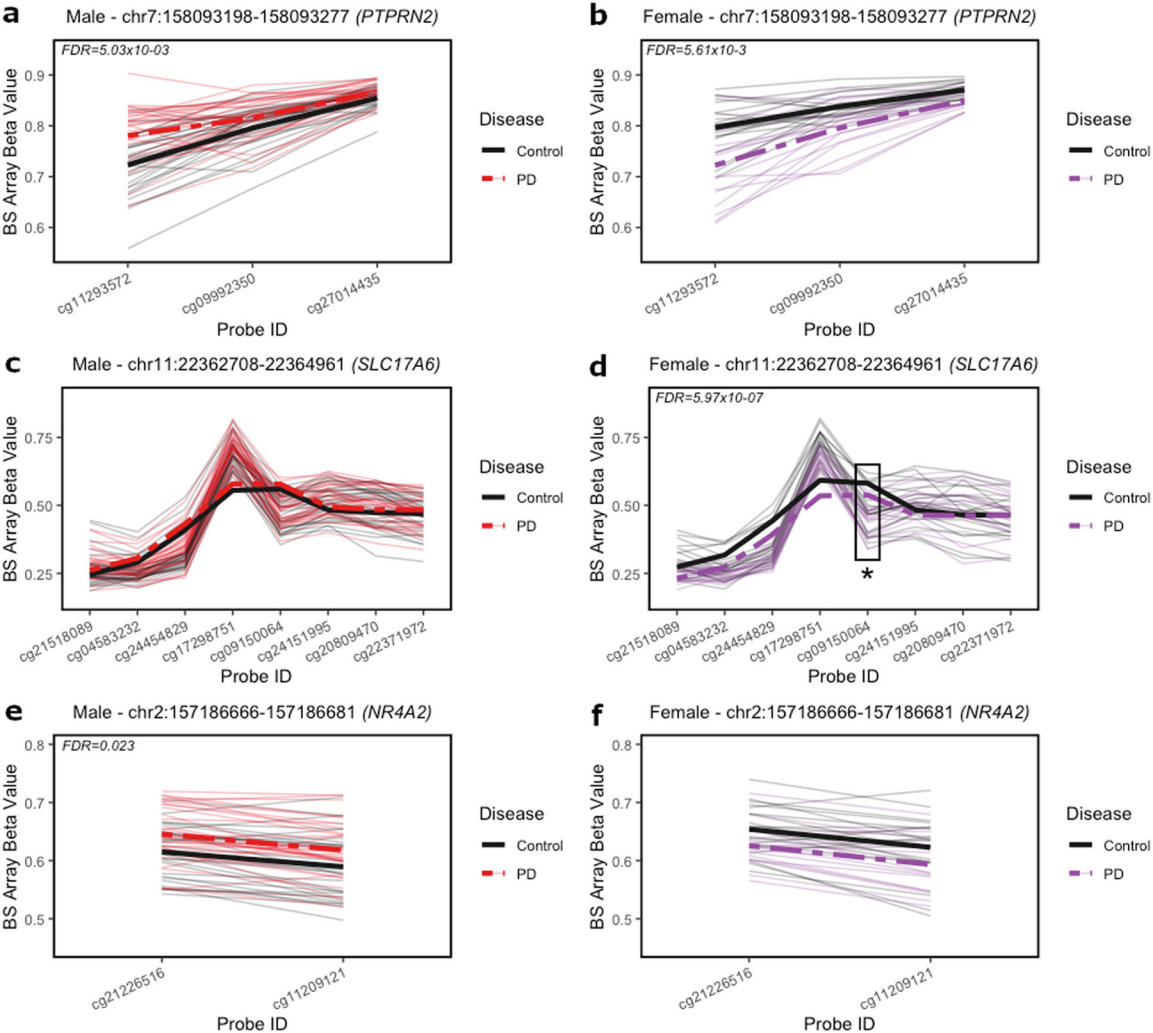
Spaghetti plots of PTPRN2, SLC17A6, and NR4A2 DMRs in males and females. Beta values (*y*-axis) represent DNA methylation across CpGs included in DMRs annotated to *PTPRN2* (chr7: 158093198–158093277), *SLC17A6* (chr11:22362708-22364961), and *NR4A2* (chr2:157186666-157186681). **a** In males, PD brains (red) exhibited significant hypermethylation at *PTPRN2* compared to control (black). **b** In females, PD brains (purple) exhibited significant hypomethylation at *PTPRN2* compared to control (black). **c** In males, PD brains (red) did not exhibit significant differential methylation at *SLC7A6* compared to control (black). **d** In females, PD brains (purple) exhibited significant hypomethylation at *SLC17A6* compared to control (black). Significant DMC (cg09150064) indicated with box and asterisk. **e** In males, PD brains (red) exhibited significant hypermethylation at *NR4A2* compared to control (black). **f** In females, PD brains (purple) did not exhibit significant differential methylation compared to control (black). Significant DMRs are indicated with minimum smoothed FDR values < 0.05. Thick solid and dashed lines represent smoothed means by group across DMRs.

**Fig. 4:**
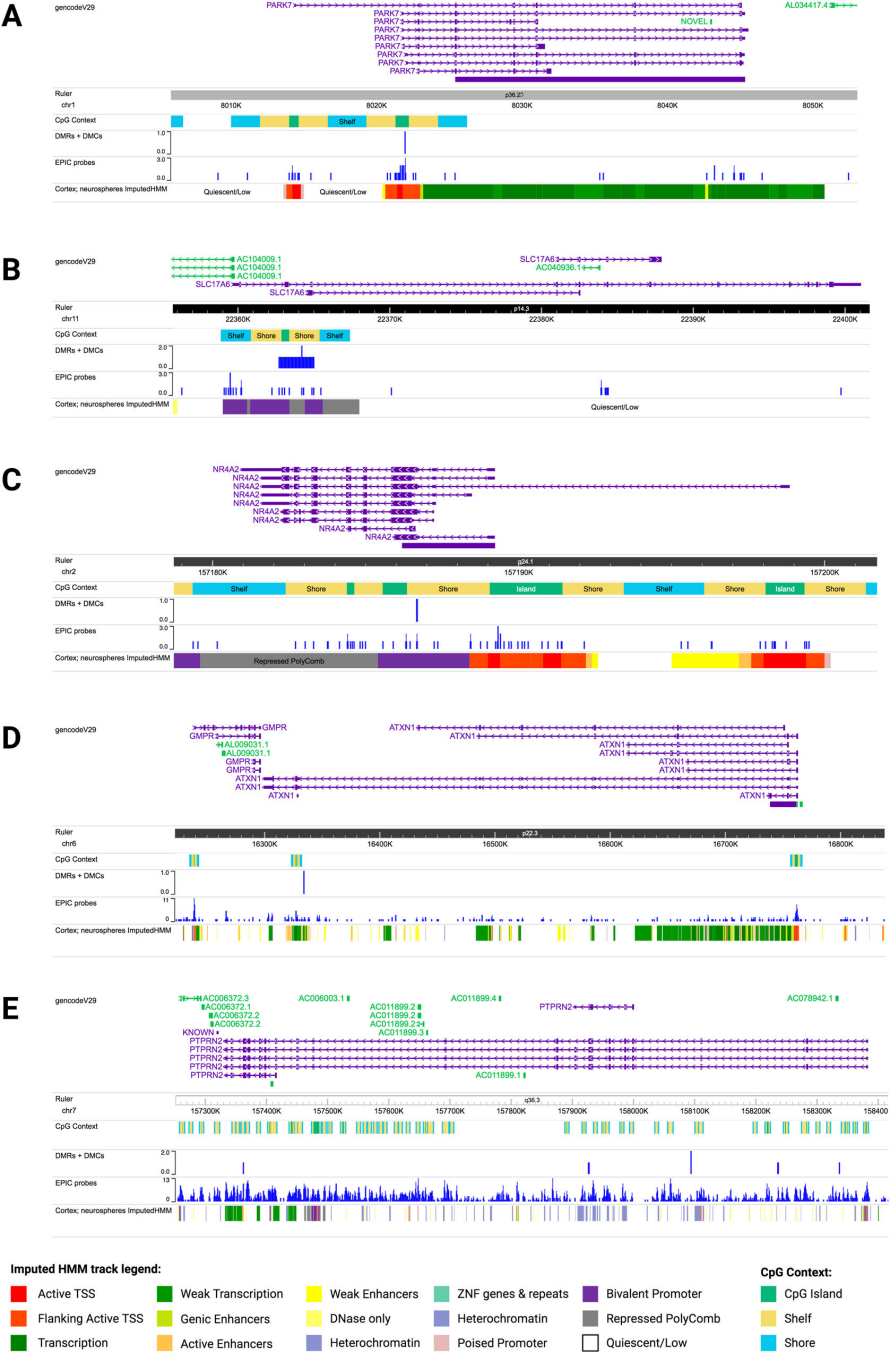
Visualization of targets genes in the Wash U Epigenome Browser. Selected genes containing PD-associated DMCs and DMRs were visualized in the Wash U Epigenome Browser. Tracks included, from top to bottom: Gencode gene annotation, chromosomal location ruler, CpG context, DMCs and DMRs, location of EPIC probes, and chromatin state (NIH Roadmap Epigenomics chromHMM imputation for cortical neurospheres). **a** *PARK7*: A single DMC was identified in the predicted transcription start site. **b** *SLC17A6*: The identified DMR overlaps with a predicted bivalent promoter and inactive repressed polycomb region, while the DMC is located within the predicted repressed polycomb region. **c** *NR4A2*: A DMC was identified within a predicted bivalent promoter. **d** *ATXN1*: The identified DMC is located within an enhancer associated with weak transcription. **e** *PTPRN2*: Multiple DMC/DMRs were identified in this gene, with one located within the transcription start site and others within heterochromatin or areas of low activity^49,50^.

Comparing these two lists of DMR by chromosomal location, 5 regions showed at least partial overlap between the two sexes (Table 2), and one region—annotated to the *PTPRN2* gene—showed exact, complete overlap in both males and females (Fig. 3a, b). This region of complete overlap (chr7:158093198–158093277) includes 3 CpGs—cg11293572, cg09992350, and cg27014435 – and is annotated to an intronic region within the *PTPRN2* gene body (Fig. 4e)^49,50^. Although this region was a DMR in both males and females, it was hypermethylated in male brains and hypomethylated in female brains, so we have highlighted this gene (Table 2, Fig. 3). Additional DMCs were also identified with the *PTPRN2* gene body (Fig. 4e)^49,50^. The other 4 regions of partial overlap – annotated to the *ZIC1, GALNT15, KIAA0040*, and *GFPT2* genes – also showed opposite relationships between PD status and DNA methylation in males and females (Table 2).

**Table 2:** Differentially methylated regions (DMRs) by Parkinson’s disease.

	Position	Annotated genes	CpG sites (n)	Mean difference in proportion methylated in PD vs. control	Min. smoothed FDR *q*-value
Male	chr3:147122664-147123477	*ZIC1, ZIC4*	8	0.042	5.36E−04
	chr3:16216606-16217127	*GALNT15*	4	−0.026	8.15E−03
	chr1:175161526-175163165	*KIAA0040*	19	−0.015	2.81E−07
	chr5:179740743-179741120	*GFPT2*	4	0.143	1.92E−02
	chr7:158093198-158093277	*PTPRN2*	3	0.029	5.03E−03
Female	chr3:147121892-147125287	*ZIC1, ZIC4*	21	−0.062	8.45E−11
	chr3:16216045-16216733	*GALNT15*	8	0.032	2.22E−05
	chr1:175161526-175162553	*KIAA0040*	18	0.021	2.23E−05
	chr5:179740188-179741120	*GFPT2*	5	−0.194	4.29E−04
	chr7:158093198-158093277	*PTPRN2*	3	−0.039	5.61E−03

Five significant DMRs (minimum smoothed FDR < 0.05) showed at least partial overlap in both sexes, as shown in the sex-stratified tables (Top = males; bottom = females). One region—annotated to the *PTPRN2* gene—showed exact, complete overlap in both males and females. All regions of overlap showed sex-specific directions of differential DNA methylation with PD. DMR modeling by Parkinson’s disease status included age and estimated glial cell proportion as covariates.

*FDR* false discovery rate.

In addition to overlapping DMRs, we also identified a female-specific DMR annotated to the *SLC17A6* gene, which included the DMC identified at this gene (Figs. 3d, 4b)^49,50^. We highlight this gene since it has previously been implicated in PD and was identified as containing most significant female-specific DMC (Fig. 2c, d). This DMR (chr11:22362708–22364961) spans a CpG island and chromatin annotated as a bivalent promotor region (Fig. 4b)^49,50^. The *SLC17A6* DMR was not significant in males (Fig. 3c).

We also identified a male-specific DMR annotated to the *NR4A2* gene (Fig. 3e), which we previously identified as a gene of interest for PD risk in a mouse model of developmental pesticide exposure^51^. This DMR (chr2:157186666–157186681) includes 2 CpGs—cg21226516 and cg11209121—that are located at an exon-intron boundary in the gene body of *NR4A2* and within chromatin annotated as bivalent chromatin (Fig. 4c)^49,50^. The *NR4A2* DMR was not significant in the female data (Fig. 3f). These results underscore that PD-related changes in cortical neuron DNA methylation are sex-specific.

### Gene ontology pathway enrichment

**Fig. 5:**
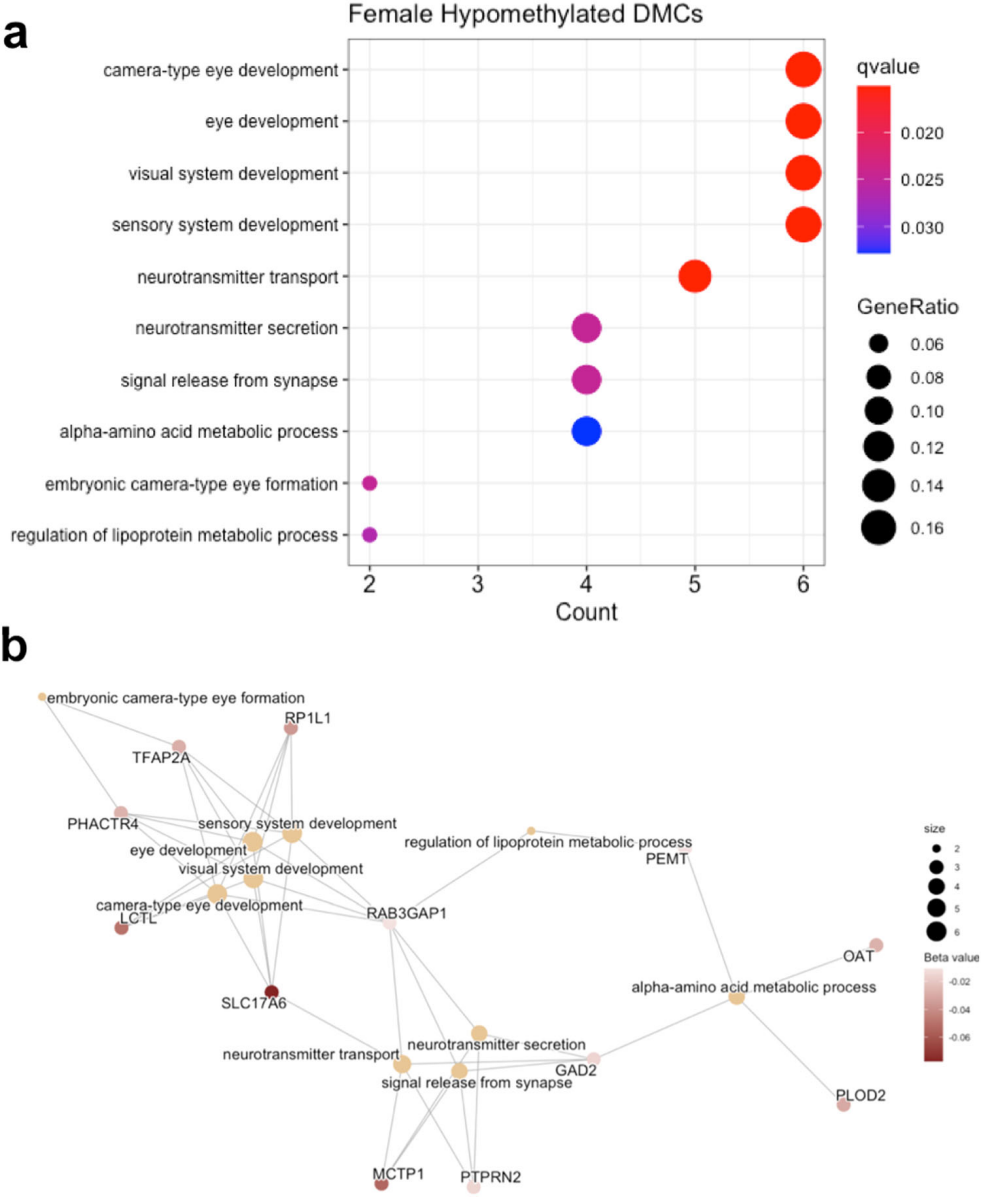
GO Term enrichment dot plot and gene-concept network plot for female hypomethylated DMCs. **a** Female hypomethylated DMCs showed enrichment for 10 pathways, including neurotransmitter transport, neurotransmitter secretion, and signal release from synapse. The *x*-axis is the number of genes in each pathway, color is used to represent FDR *q*-value (*q*-value), and size is used to represent GeneRatio, the ratio of differentially methylated genes in each GO term to the total number of genes input into the hypergeometric test. **b** Several differentially methylated genes were included in the enriched pathways, including *PTPRN2*, which also showed differential DNA methylation at the region level in both males and females. The size of each node is used to represent the number of genes in each GO term, and color represents the magnitude of decrease in DNA methylation (beta value) for each annotated probe. Connections between genes and GO terms represent inclusion of the gene in that GO term.

The *clusterProfiler* R package was used to determine whether DMCs annotated to genes enriched for specific gene ontology biological process (GOBP) pathways. GOBP pathway enrichment analysis was not performed on male DMCs due to the low number of significant CpGs (*n* = 3) but was performed on the genes annotated to female hypomethylated DMCs and hypermethylated DMCs separately. Hypo- and hypermethylated DMCs were considered separately due to expected differences in associations between increased or decreased DNA methylation and gene regulation. Female hypermethylated DMCs did not show enrichment for any pathways, but female hypomethylated DMCs showed enrichment for 10 pathways, including several developmental pathways, neurotransmitter transport, neurotransmitter secretion, and signal release from synapse (Fig. 5a). Several differentially methylated genes were included in these GO term pathways, including *SLC17A6* and *PTPRN2* (Fig. 5b), the latter of which showed differential DNA methylation at the regional level in both males and females.

The *clusterProfiler* R package was also used to determine whether DMRs annotated to genes enriched for specific gene ontology biological process (GOBP) pathways. In both males and females, hypermethylated DMRs did not show any enrichment for GOBP terms. Meanwhile, for the hypomethylated DMRs, females showed significant enrichment for 6 GOBP terms (*q*-value < 0.05), including cell fate commitment and glucose homeostasis (Additional File 5). These enriched pathways for the female hypomethylated DMRs are collectively related to cell fate and metabolism. In male hypomethylated DMRs, 4 pathways approached significance (*q*-value = 0.06), including semaphorin-plexin signaling pathways involved in axon guidance and neuron projection guidance (Additional File 5).

### Epigenetic clock analysis

**Fig. 6:**
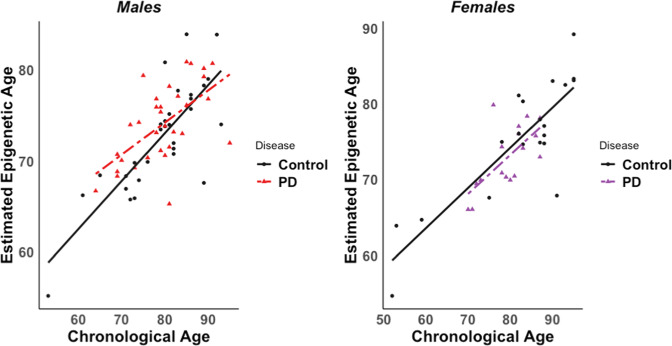
Scatterplots of male and female estimated epigenetic age vs. chronological age at death. Estimated epigenetic age showed a significant positive association with chronological age in males (left) and females (right). However, PD status did not significantly modify trajectories of epigenetic age. Control samples are shown as black dots, and PD samples are shown as red or purple triangles for males and females, respectively.

In our epigenetic clock analysis, estimated epigenetic age significantly predicted chronological age in both sexes, with estimated age (“EpiAge”) significantly associated with chronological age at death (males: Beta coefficient = 1.1929, *p*-value = 8.97 × 10^−9^; females: Beta coefficient = 1.3515, *p*-value = 2.51 × 10^−9^). These results confirm that this is a well-calibrated epigenetic clock where there was an approximate 1 unit increase in estimated epigenetic age with a 1 unit increase in chronological age (Table 3). However, in our data, PD status did not modify trajectories of epigenetic aging in either sex (Fig. 6, Table 3).

**Table 3:** Epigenetic clock linear regression results.

Coefficients:	Estimate	Std. error	*t*-value	*P*-value
Combined data (*N* = 99)				
EpiAge	1.273	0.1168	10.897	<2E−16
Disease PD	22.7787	16.8207	1.354	0.179
EpiAge:DiseasePD	−0.3206	0.2278	−1.407	0.163
Male Data (*n* = 62)				
EpiAge	1.1929	0.1778	6.711	8.97E−09
Disease PD	11.8959	22.137	0.537	0.593
EpiAge:DiseasePD	−0.1788	0.3004	−0.595	0.554
Female Data (*n* = 37)				
EpiAge	1.3515	0.1672	8.081	2.51E−09
Disease PD	35.7668	28.8822	1.238	0.224
EpiAge:DiseasePD	−0.484	0.3927	−1.233	0.226

Models were run for male subjects (top), and female subjects (bottom). EpiAge refers to estimated epigenetic age. DiseasePD refers to the PD status variable (reference group = control). EpiAge:DiseasePD is the interaction term between estimated epigenetic age and PD status that determines whether PD brains exhibit altered trajectories of epigenetic age compared to control. Estimate = beta coefficients from regression model.

*Std. Error* standard error.

## Discussion

In this study, we identified sex-specific, PD-associated DNA methylation changes in a total of 434 unique genes, including *PARK7* (DJ-1), *SLC17A6* (VGLUT2), *PTPRN2* (IA-2β), *NR4A2* (NURR-1), as well as other genes involved in developmental pathways, neurotransmitter packaging, and release, and axon/neuron projection guidance (Figs. 2–5). Our data did not show accelerated epigenetic aging in PD (Fig. 6, Table 3). This study expands on the existing literature through the utilization of nuclei-sorted cortical neurons, the inclusion of both sexes, and estimation of epigenetic age.

### Sex-specificity of PD-associated differential DNA methylation

Sex is known to have significant effects on the epigenome, and recent work has shown sex-specific directionality of associations between DNA methylation and Alzheimer’s disease, suggesting that sex might also affect the relationship DNA methylation and other neurodegenerative diseases^52^. Given this fact, as well as the known sex differences in susceptibility and progression of PD by sex, we chose to stratify our analysis by sex, providing a genome-wide study of DNA methylation in PD that analyzed male and female samples separately^29–31^. To confirm that sex stratification was preferred, we compared *p*-value histograms between sex-adjusted and sex-stratified models, showing that the sex-stratified models provided more appropriate histograms (uniform with an overabundance of low *p*-values), whereas the combined model produced a non-uniform histogram with a skew towards high *p*-values (Supplementary Figure 2). This underscores the importance of stratifying data by sex rather than including as a covariate during analysis. While we expected to identify differences in DNA methylation in male and female samples, the near-total lack of overlap between sexes was striking, suggesting that epigenetic regulation may underlie the well-recognized sex differences in PD susceptibility and progression.

### Male-specific PD-associated DNA hypomethylation at the PARK7 locus

The most significant DMC in males showed PD-related hypomethylation and was located in the *PARK7* locus within a CpG island that spans the transcription start site (Figs. 2, 4). Mutations in the *PARK7* locus, which encodes the DJ-1 protein, cause autosomal recessive, early-onset Parkinson’s disease and oxidized DJ-1 has been observed in brains of idiopathic PD patients^53,54^. Existing research shows that DJ-1 has multiple known neuroprotective functions—transcriptional regulator, molecular chaperone, and antioxidant—and downregulation of this locus is thought to play a role in both familial and idiopathic forms of PD^55–58^. Thus, it is possible that epigenetic regulation of this locus modifies expression in idiopathic PD.

Two previous studies examining the effects of PD status on DNA methylation at the *PARK7* locus failed to identify a significant relationship^59,60^. However, these studies used bisulfite pyrosequencing to measure DNA methylation at regions in the *PARK7* locus that do not overlap our DMC. In addition, one study was performed in blood leukocytes, which may not reflect DNA methylation in brain tissue. The other utilized a very small sample size (*n* = 5 per group) and used bulk postmortem brain tissue samples from substantia nigra, parietal cortex, and occipital cortex^59,60^. Given these differences, the existing data do not necessarily contradict our *PARK7* results.

### Female-specific PD-associated DNA hypermethylation at the ATXN1 locus

The most significant DMC in female subjects was located in a weak enhancer region within the *ATXN1* gene, which showed PD-related hypermethylation (Figs. 2, 4). *ATXN1* encodes Ataxin-1 (ATXN1), which is involved in transcriptional repression of a large number of target genes and is necessary for normal brain development and function^61,62^. Expanded polyglutamine repeats in this gene cause spinocerebellar ataxia type 1 (SCA1)^61,63^. Although the *ATXN1* gene has not been previously studied in the context of PD, our data showing altered epigenetic regulation of the *ATXN1* gene in PD brains opens new avenues of research. It is possible that epigenetic regulation at this locus leads to changes in the expression of *ATXN1* and its target genes, affecting the susceptibility of cortical neurons to PD-related dysfunction and pathology.

### Male-specific PD-associated changes in DNA methylation at NR4A2

In males, we also identified PD-related hypermethylation in an exon just downstream of an exon-intron boundary in the gene body of *NR4A2* (Figs. 3, 4). *NR4A2* encodes the nuclear receptor related-1 (NURR1) protein, a transcription factor critical for dopaminergic neuron development and maintenance. Previous research suggests that downregulation of the *NR4A2* gene in the brain may contribute to PD pathogenesis^64–66^. Supporting this idea, in a previous animal model study, we showed that developmental exposure to the organochlorine pesticide dieldrin, which is known to be associated with increased PD risk, led to sex-specific changes in DNA methylation in the *Nr4a2* gene body in mice^51^. These previous results, along with our present data, suggest that sex-specific epigenetic regulation at the *NR4A2* gene may play a role in idiopathic PD risk.

### Female-specific PD-associated DNA hypomethylation at the SLC17A6 locus

In females, we identified PD-related hypomethylation within the *SLC17A6* gene body (Figs. 2–4). *SLC17A6* encodes the vesicular glutamate transporter 2 (VGLUT2), which has been implicated in PD pathogenesis via multiple lines of evidence^67^. Studies using in vivo and in vitro models show increased VGLUT2 expression in dopaminergic (DA) neurons in response to the neurotoxicants, rotenone, 6-OHDA and MPP^+^^68,69^. Further, deletion of VGLUT2 in mice exacerbates the neurotoxic effects of 1-methyl-4-phenyl-1,2,3,6-tetrahydropyridine (MPTP) exposure^70^. Additional studies suggest that neurons that co-express VGLUT2 and VMAT2 show differentially vulnerability in PD and differences in neuronal structure^71,72^. Our analysis did not assess DNA methylation in DA neurons of the nigrostriatal pathway, which have been the subject of much of this work on VGLUT2 in the context of PD. However, the observed hypomethylation in the *SLC17A6* gene in the parietal cortex suggests that this locus could show altered epigenetic regulation of gene expression during disease progression. Given the known neuroprotective role for VGLUT2, future studies should examine whether epigenetic changes at *SLC17A6* are associated with decreased VGLUT2 expression and increased neuronal susceptibility.

### Sex-specific PD-associated changes in DNA methylation at PTPRN2

In our regional analysis, one DMR—annotated to the *PTPRN2* gene—showed exact, complete overlap in both males and females (Table 2). Although the identified *PTPRN2* DMR was differentially methylated in both males and females, it was hypermethylated in brains from male PD patients and hypomethylated in brains from female PD patients (Fig. 3). *PTPRN2* encodes Protein Tyrosine Phosphatase Receptor Type N2 (IA-2β), which is expressed on dense core and synaptic vesicles. Altered methylation of this gene has been previously implicated in PD in a study of longitudinal blood samples that showed an association between hypomethylation of a different CpG within the *PTPRN2* gene in whole blood and faster motor progression in PD^73^. In addition, deletion of this gene and the related IA-2 protein in mice leads to reduced levels of norepinephrine, dopamine (DA) and serotonin in the brain and decreased release of DA, gamma-aminobutyric acid (GABA), and glutamate from synaptosomes^74^. One previous study found decreased expression of *PTPRN2* in the SN of PD patients, while another found increased expression in DA neurons from PD patients with *LRRK2* G2019S mutations^75,76^. Interestingly, given the known role of environmental exposures in PD and work in our lab investigating pesticide exposures and PD^51,77^, another study found an association between pesticide exposure and hypomethylation at a CpG in *PTPRN2* in blood^78^. The opposite directionality of the relationship between *PTRPN2* DNA methylation and PD status in males and females suggests that differential epigenetic regulation may be important in sex differences in PD.

### Sex-specific pathways enriched for DNA methylation changes

In addition to our gene-level differences, pathway analysis of differentially methylated genes identified distinct enriched pathways in male and female subjects (Fig. 5). Female-specific differentially methylated genes were enriched for pathways including neurotransmitter transport, neurotransmitter secretion, and signal release from synapse (Fig. 5c). These pathways were not enriched in differentially methylated genes identified in male subjects. Instead, the semaphorin-plexin signaling pathways involved in axon guidance and neuron projection guidance were enriched in male-specific differentially methylated genes. These pathways included *PLXNB1, PLXNC1*, and *PLXNB3*, three genes that encode proteins in the plexin family, which act as transmembrane receptors for the semaphorins. The semaphorin-plexin signaling pathway is a key regulator of morphology and motility in many different cell types, including those that make up the nervous system, and is known to play a role in axon guidance^79,80^. As with our gene-specific results, the sex-specificity of these networks is striking and reinforces that epigenetic regulation in PD is highly sex-specific and may contribute to the known sex differences in PD.

### PD is not associated with accelerated epigenetic aging

Over the past decade, recognition has been growing that the epigenome, especially DNA methylation, can be used to closely predict chronological age^41,81–83^. Based on this, researchers have established epigenetic clocks that use loci with the most predictable DNA methylation levels to provide estimates of epigenetic (or biological) age^81^. In a previous study using one of these epigenetic clocks, researchers found accelerated epigenetic aging in PD blood samples compared to control^45^. However, to our knowledge, no studies have tested whether epigenetic aging is accelerated in PD brain tissue. Here, we used an epigenetic clock specifically designed for human cortical tissue to estimate epigenetic age of control and PD parietal cortex samples^84^. In our analyses, we showed that our epigenetic clock was well-calibrated to predict chronological age at death, but found no evidence of significant epigenetic age acceleration by PD status in our cohort (Fig. 6). These data suggest that accelerated epigenetic aging may not play a role in PD development. Alternatively, the lack of relationship between epigenetic aging and disease status could also be a byproduct of the selected brain region—parietal cortex—which shows effects of PD pathology very late in disease. In contrast, neurons from brain regions that are impacted early in PD (i.e. midbrain) might demonstrate positive relationships between epigenetic aging and disease. Furthermore, the epigenetic aging algorithm used in this study was designed for human cortical tissue, but not specifically for neurons from parietal cortex, which could inflate the variability of our epigenetic age estimates and limit our ability to detect significant associations. Lastly, it is also possible that an association between disease and epigenetic age could occur in middle-age, but disappear at late ages, as has been shown previously for obesity-related epigenetic aging^85^. Samples from PD and control subjects across a broader range of ages would be needed to test this hypothesis.

## Conclusions

Our study provides a genome-wide analysis of DNA methylation in an enriched neuronal population from both male and female PD brain tissue. Despite our study design’s strengths, there remain some limitations in our analysis that create opportunities for future studies. First, our results do not distinguish between DNA methylation and DNA hydroxymethylation, which show distinct genomic distributions and associations with gene expression in the brain, as well as differential responses to environmental exposures^9,86,87^. As such, DNA methylation and DNA hydroxymethylation may play discrete roles in neurodegenerative diseases^9,87,88^. It is critical for future studies to use modifications to the bisulfite conversion protocol, like oxidative BS or enzymatic conversion methods, that can distinguish between these marks. Second, because we used the Illumina EPIC array in this study, our data did not provide complete genomic coverage across the identified target genes. As a result, future work must follow up on these data with loci-specific analysis using BS-pyrosequencing or other targeted approaches to assess all cytosines at the identified loci. Third, the EPIC array does not provide a true “genome-wide” wide analysis of DNA methylation. This array was designed largely for cancer research, not brain-specific and neurodegenerative disease studies, and many genomic regions of interest to the PD field are not covered on the array. True genome-wide analysis requires whole-genome bisulfite sequencing (WGBS), but WGBS remains cost-prohibitive to scale up for a large cohort, making it unfeasible for this project. Although our study had a sample size smaller than recent recommendations published by Mansell et al after sex stratification, we experimentally controlled for multiple variables, including sex, race, and age, which were included as covariates in their estimates^48^. This increases our statistical power relative to the proposed minimum sample size, but future studies should include larger cohorts or be combined with publicly available data. Finally, we only analyzed the neuron-enriched population from our MACS-sorted samples. Given the growing body of evidence supporting the importance of non-neuronal cell types in PD, future work will aim to carry out similar analyses in neuron-depleted samples.

Despite our experimental limitations, this study provides a genome-wide analysis of DNA methylation in enriched neurons from male and female human postmortem brain tissue. Results from this work support the idea that epigenetic regulation is an important mechanism in PD pathogenesis and point to specific genes and pathways for further study.

## Methods

### Magnetic-activated cell sorting

De-identified parietal cortex samples were obtained from BSHRI. NeuN-positive (NeuN^+^) nuclei were enriched from 100 mg of flash frozen parietal cortex tissue using a two-stage magnetic-assisted cell sorting (MACS) method. First, 100 mg of frozen tissue were briefly thawed on ice and homogenized in a 2 mL, 1.4 mm ceramic bead tube (Thermo Fisher Scientific, Cat. # 15–340–153) with 1 mL of Nuclear Extraction Buffer (NEB) for 10 s at 4 m/s. NEB consisted of 0.32 M sucrose, 0.01 M Tris-HCl pH 8.0, 0.005 M CaCl_2_, 0.003 M MgCl_2_, 0.0001 M EDTA, and 0.1% Triton X-100, up to a stock volume of 1 L using water. Immediately prior to use, 0.001 M DTT was added to NEB. Homogenized samples were loaded into a 13 mL ultracentrifuge tube (BeckmanCoulter, Cat. # 331372) with 4 mL of NEB. Using a glass pipette, 7 mL of sucrose solution was pipetted down the side of each sample tube to create a sucrose gradient. Sucrose solution consisted of 1.8 M sucrose, 0.01 M Tris-HCl pH 8.0, 0.003 M MgCl_2_, up to a stock volume of 1 L using water. Immediately prior to use, 0.001 M DTT was added to NEB. After addition of sucrose, samples were spun at 4 °C, 24,000 rpm in the Sorvall Wx+ Ultracentrifuge in a swing bucket rotor (TH-641). Once the centrifugation was complete, the supernatant and debris layer found at the concentration gradient were both removed with the use of a vacuum, while being careful not to disturb the pellet containing the nuclei at the bottom of the tube. Next, 1 mL of primary antibody (anti-Neun 488—Millipore, Cat. # MAB377X) in MACS buffer was added to each nuclei pellet and placed on ice for 10 min. MACS buffer consisted of 0.5% Bovine Serum Albumin solution (Sigma-Aldrich, Cat. # A1595) in PBS pH 7.2 (Gibco, Cat. # 20012-027). Samples were then mechanically pipetted up and down 10–15 times to completely dissolve the nuclei pellet within the primary antibody-MACS buffer solution. This solution of nuclei was then transferred to a 2 mL tube and incubated for 60 min at 4 °C. After incubation, 40 µL of MACS Microbeads (anti-mouse IgG Microbeads - Miltenyi, Cat. # 130-048-401) were added to each sample. Samples were then inverted 4–5 times and incubated at 4 °C for 30 min. After incubation, nuclei were centrifuged at 300*g* for 10 min. Supernatant was then removed, and the nuclei were resuspended in 2 mL of MACS buffer and transferred to a MACS MS column (MS Separation columns—Miltenyi, Cat. # 130-042-201) that was pre-washed with MACS buffer and attached to the Miltyeni OctoMACS™ Separator. Positive selection of NeuN^+^ cells was then performed according to the standard MACS MS Columns protocol available from Miltenyi Biotec. After the first round of magnetic separation, NeuN^+^ nuclei were run through a separate, second MACS MS column to maximize cell type enrichment.

### Enrichment validation using flow cytometry

Isolated nuclei were analyzed for flow cytometry on a CytoFlex S (Beckman Coulter), and data were analyzed using FlowJo V10. Single nuclei were identified by the presence of DAPI staining in a plot of 405–450/45-width signal vs 405–450/45-area signal. These nuclei were then analyzed for NeuN expression by looking for A488 fluorescence in the 488–525/40 channel. Determination of A488 positivity was made by comparing to a sample stained only with DAPI to define the background. Percent positivity for each sample was defined as the percentage of events in the NeuN-A488 + gate, divided by the total number of events identified as Nuclei.

### DNA extraction, bisulfite treatment, and EPIC arrays

DNA was isolated from enriched NeuN^+^ nuclei using the Qiagen QIAamp DNA Micro Kit (Cat. # 56304), with some modifications to maximize yield. Given that samples were already homogenized during nuclei isolation, the sample lysis and incubation steps of the QIAamp DNA Micro Kit protocol were removed. Instead, 20 uL of proteinase K were added directly to each MACS eluate. Samples were then vortexed for 15 seconds and incubated at room temperature for 15 min. In addition, the optional carrier RNA was added to Buffer AL, the incubation time after the addition of 100% ethanol was increased to 10 min, the incubation time for the elution buffer was increased to 5 min, and the final elution step was repeated using 10 mM Tris-HCl pH 8.0.

Intact genomic DNA yield was quantified by Qubit fluorometry (Life Technologies). Bisulfite conversion was performed on 500 ng genomic DNA using the TrueMethyl Array kit (Cambridge Epigenetix). All conversion reactions were cleaned using SPRI-bead purification and eluted in Tris buffer. Following elution, BS-converted DNA was denatured and processed through the EPIC array protocol. The EPIC array contains ~850,000 probes that query DNA methylation at CpG sites across a variety of genomic features, including CpG islands, RefSeq genic regions, ENCODE open chromatin, ENCODE transcription factor binding sites, and FANTOM5 enhancer regions. To perform the assay, converted DNA was denatured with 0.4 N sodium hydroxide. Denatured DNA was then amplified, hybridized to the EPIC bead chip, and an extension reaction was performed using fluorophore-labeled nucleotides per the manufacturer's protocol. Array BeadChips were scanned on the Illumina iScan platform.

### EPIC array data processing

After scanning on the iScan platform, BeadChip IDAT files were imported into R and processed using an in-house bioinformatics pipeline. This pipeline combined the *minfi* (version 1.22.1), *ChAMP* (version 2.14.0), *posibatch* (version 1.0), *ENmix* (version 1.12.4), *CETS* (version 3.03), and *dplyr* (version 1.0.8) packages in R. Quality control was assessed for internal control probes using the *ENmix* plotCtrl function. Failed probes were identified as those with a detection p-value > 0.01 in any sample. Failed probes were then removed from downstream analyses when detection p-value was > 0.01 in >5% of samples. No samples exceeded the selected threshold of failed probes (>10%) to warrant removal prior to analysis. Cross-reactive probes and probes containing SNPs were removed based upon previous identification^89,90^. After removal of technical artifacts, dye-bias correction was performed with *ssNoob* within *minfi*^91^. The proportion of neuronal vs. glial cells in each sample was estimated with *CETS*^92^. Samples with estimated glial cell to neuronal cell proportion > 0.90 were removed from analysis. This removed one sample from the male data set, leaving *n* = 62 males. Batch effects were assessed using the *ChAMP* package^93^, and then a beta value matrix for all samples was corrected for batch and positional effects using the *posibatch* R package^94^. After beta value estimation, we filtered out samples with mean DNA methylation beta value < 0.01. This beta value cutoff was instituted due to increased variability and decreased interpretability of beta values at such low levels. As a final step, we removed probes that were not annotated to a gene in the Illumina EPIC array manifest to ensure the interpretability of results after modeling. After all filtering steps, 552,332 probes remained for differential DNA methylation testing in the sex-stratified datasets. Code for data processing, filtering, and modeling is available (Additional Files 6, 7, and 8).

### Statistical analysis

#### Differential testing for individual CpG sites

The generalized additive models for location, scale, and shape (*gamlss*; version 5.4-1) R package was used to test for differentially methylated CpGs (DMCs)^95^. This package provides functions to perform multivariate regression modeling while maintaining flexibility regarding term structure and distribution. For DMC testing, we assumed a normal distribution (standard linear regression model) and included both the mu (mean) and sigma (scale parameter) in models to test for differences in the mean by disease state while accounting for potential differences in variability. Linear regression was used in lieu of negative-binomial or beta regression modeling due to recent work showing that linear regression does not increase the likelihood of false positives in EPIC array-based epigenome-wide association studies^48^. All models were stratified by sex, and age and estimated glial cell proportion were included as covariates. Sex stratification was performed in response to a skewed, non-uniform p-value histogram that was produced by an initial model that simply included a covariate to adjust for sex (Supplementary Figure 2). The need to stratify by sex was not unexpected and this possibility was included in our preregistration plan. After stratifying for sex, p-value histograms were largely uniform, with peaks near zero (Supplementary Figure in Dryad data repository), as would be expected from well-calibrated hypothesis tests^96^.

An unadjusted *p*-value cutoff of <9 × 10^−8^ was used to assess the significance of DMCs, as recommended in a recent report on human epigenome-wide association studies^48^. All statistical models were run using R statistical software (version 4.1.0). Annotation of detected differential probes was performed using the Illumina EPIC array manifests. Code for data processing, filtering, and modeling is available as supplementary files.

#### Differential testing for genomic regions

The *DMRcate* R package (version 2.8.5) was used to test for differentially methylated regions^97^. In this package, the model matrix was established using the following code: design = model.matrix(~factor(disease) + glial_cell_proportion + age). For DMR analysis, Lamba was set to 1000 and the minimum number of CpGs was set to 2. All models were stratified by sex, and both age and estimated glial cell proportion were once again included as covariates in the modeling. The Benjamini–Hochberg False Discovery Rate (FDR) method was used to generate minimum smoothed *q*-values that account for multiple testing^98^. For the regional analysis, the significance cutoff was set at a minimum smoothed FDR *q*-value < 0.05.

#### Gene ontology pathway enrichment

The *clusterProfiler* R package (version 4.2.2) was used to determine whether differentially methylated genes were enriched for specific gene ontology biological process (GOBP) pathways^99^. Specifically, the enrichGO function was used to run hypergeometric over-representation tests on lists of differentially methylated gene IDs. Gene ID lists were split into hypermethylated DMCs or DMRs and hypomethylated DMCs or DMRs prior to analysis. The list of genes with annotated probes in the EPIC array manifest was used as the universe of total tested genes. Separate, sex-stratified analyses were performed for genes annotated to DMCs and DMRs. GOBP pathway enrichment analysis was not performed on male DMCs due to the low number of significant CpGs (*n* = 3). For enrichment analyses in *clusterProfiler*, redundant pathways were consolidated using the *simplify* function with default parameters, and significance cutoff was set at FDR *q*-value < 0.05.

#### Epigenetic clock analysis

Epigenetic age was estimated for all parietal cortex samples using a recently published algorithm designed specifically for the human cortex^84^. Estimates of epigenetic age (years) and known age at death (years) were then compared using standard linear regression with an age*disease interaction term. The interaction term was used to determine whether categorical PD status (control vs. PD) altered trajectories of epigenetic age compared to known chronological age. Epigenetic age regression modeling was performed on all data combined, as well as sex-stratified data. For these regression analyses, significance was set at *p*-value < 0.05.

## Supplementary Information

Flow cytometry plots

Male DMCs

Female DMCs

Male DMRs

Female DMRs

GOBP terms for hypomethylated DMRs

p-value histograms

R markdown for EPIC array processing

knitted html

R markdown for modeling

## Data Availability

This study was preregistered with Open Science Framework: https://osf.io/z4vbw^100^. All data acquired and analyzed for this study are available in the associated Dryad data repository: 10.5061/dryad.7d7wm37w0.
