# Supplementary figures and images for: Parkinson’s disease-associated, sex-specific changes in DNA methylation at PARK7 (DJ-1), SLC17A6 (VGLUT2), PTPRN2 (IA-2β), and NR4A2 (NURR1) in cortical neurons
Source: NPJ Parkinsons Dis. 2022 Sep 23;8:120. doi: 10.1038/s41531-022-00355-2 (PMC9508164; doi:10.1038/s41531-022-00355-2)

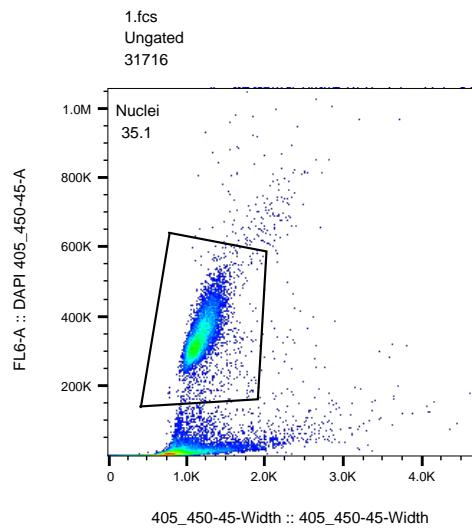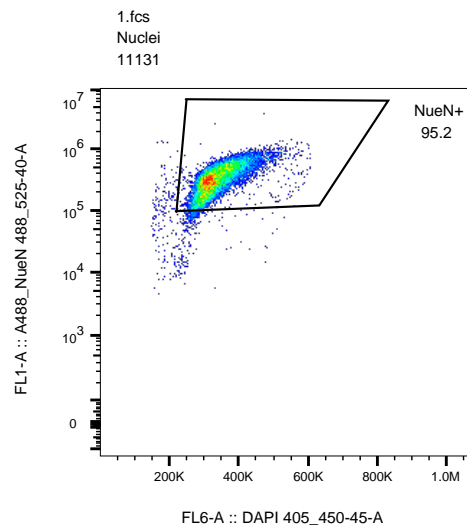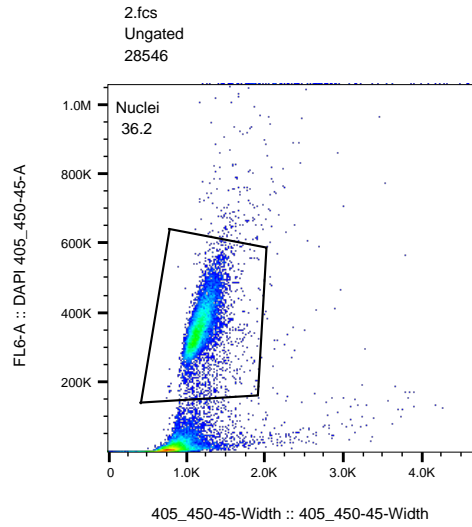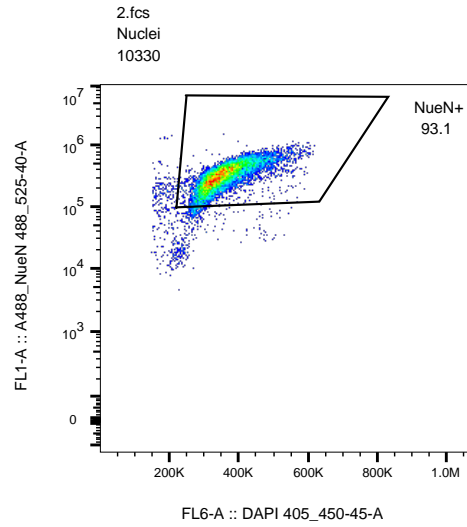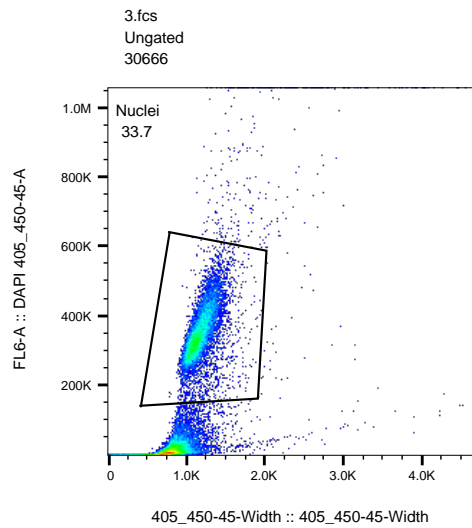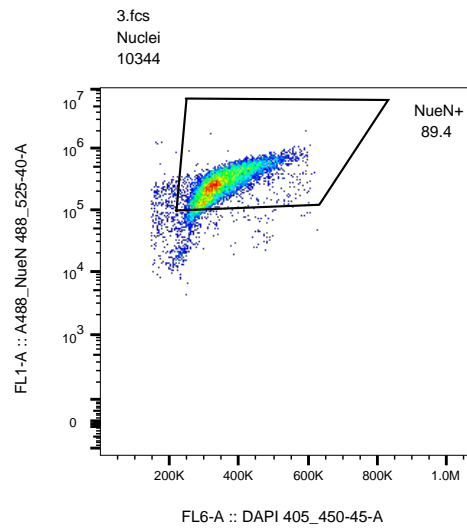

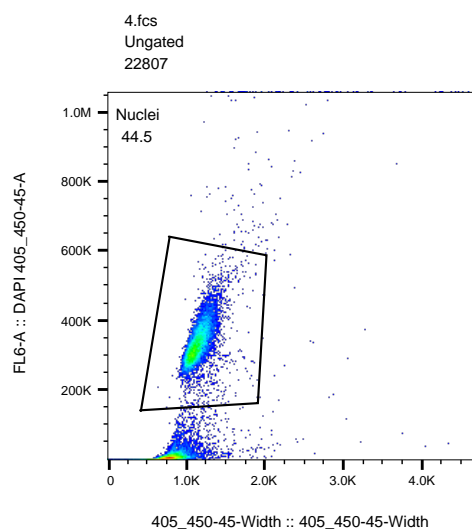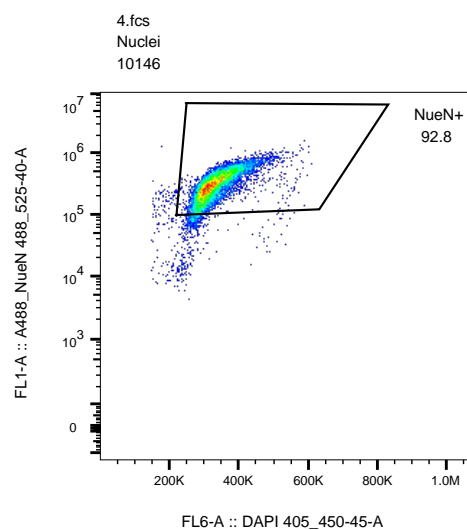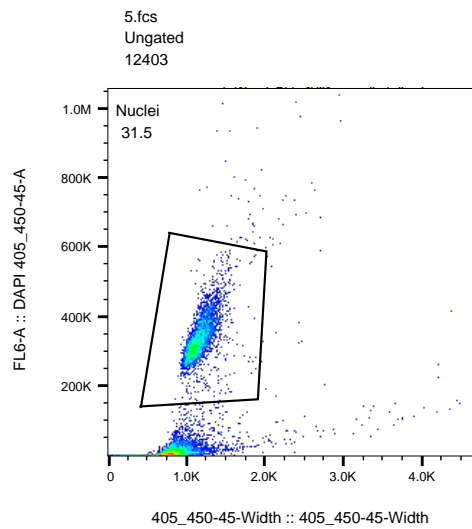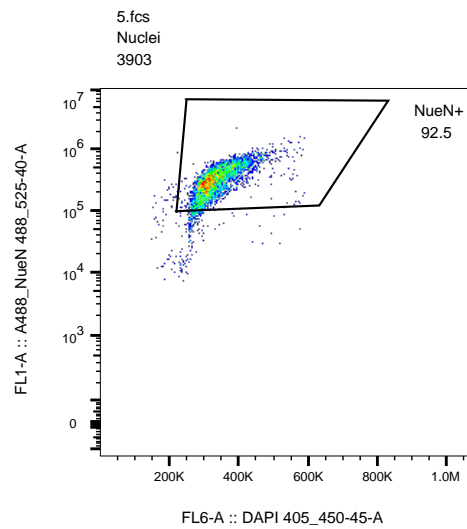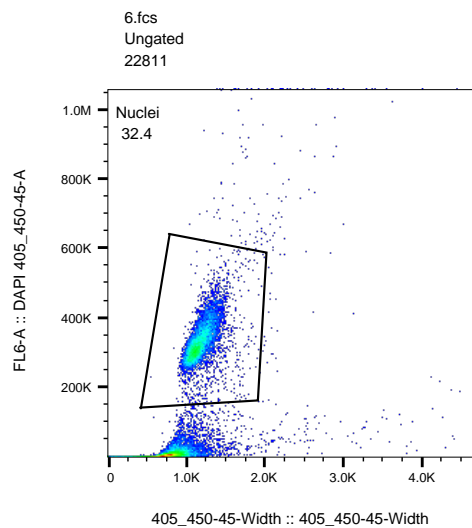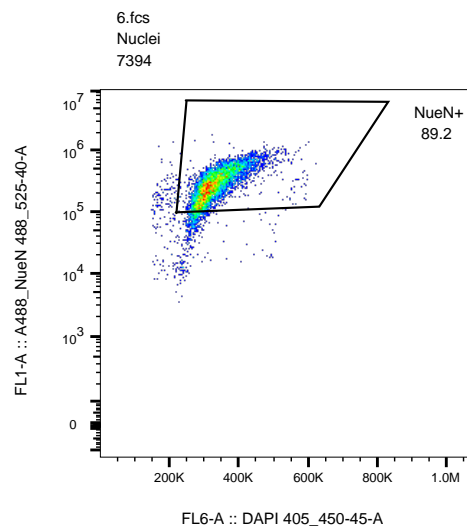

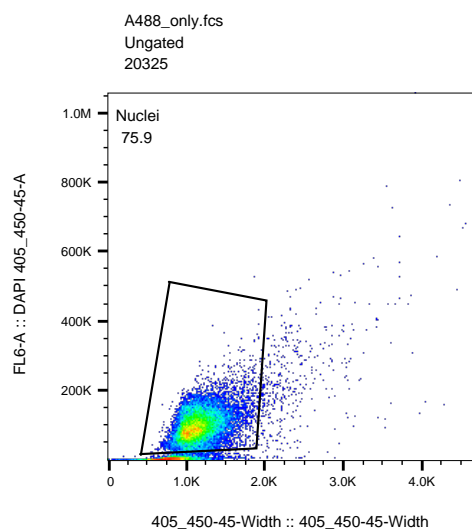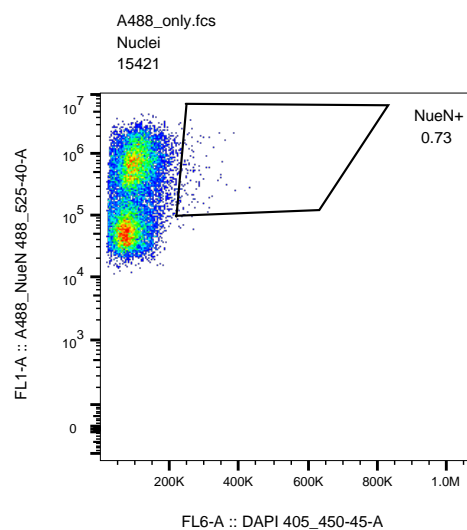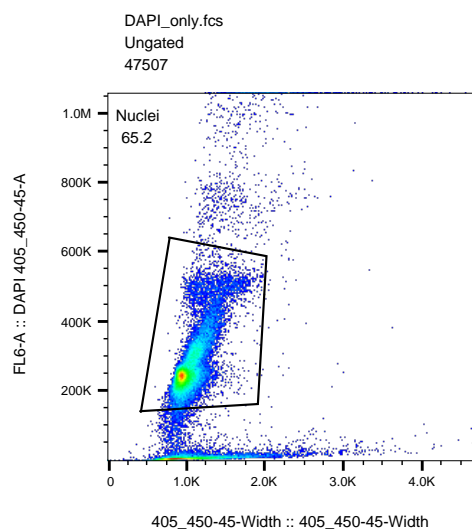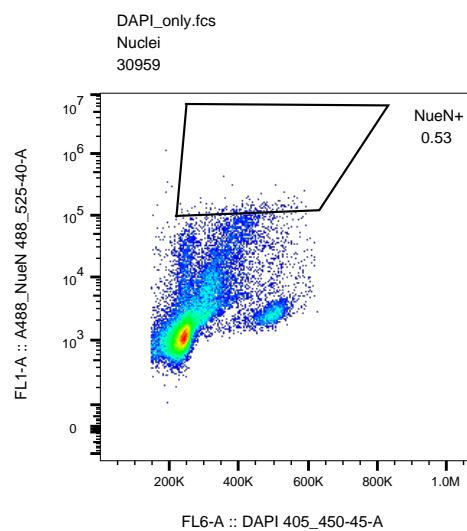

Supplement: Supplementary file 1 — Flow cytometry plots [file 41531_2022_355_MOESM1_ESM.pdf]
