# Supplementary material for: Parkinson’s disease-associated, sex-specific changes in DNA methylation at PARK7 (DJ-1), SLC17A6 (VGLUT2), PTPRN2 (IA-2β), and NR4A2 (NURR1) in cortical neurons
Source: NPJ Parkinsons Dis. 2022 Sep 23;8:120. doi: 10.1038/s41531-022-00355-2 (PMC9508164; doi:10.1038/s41531-022-00355-2)
Supplement: Supplementary file 7 — p-value histograms [file 41531_2022_355_MOESM7_ESM.pdf]

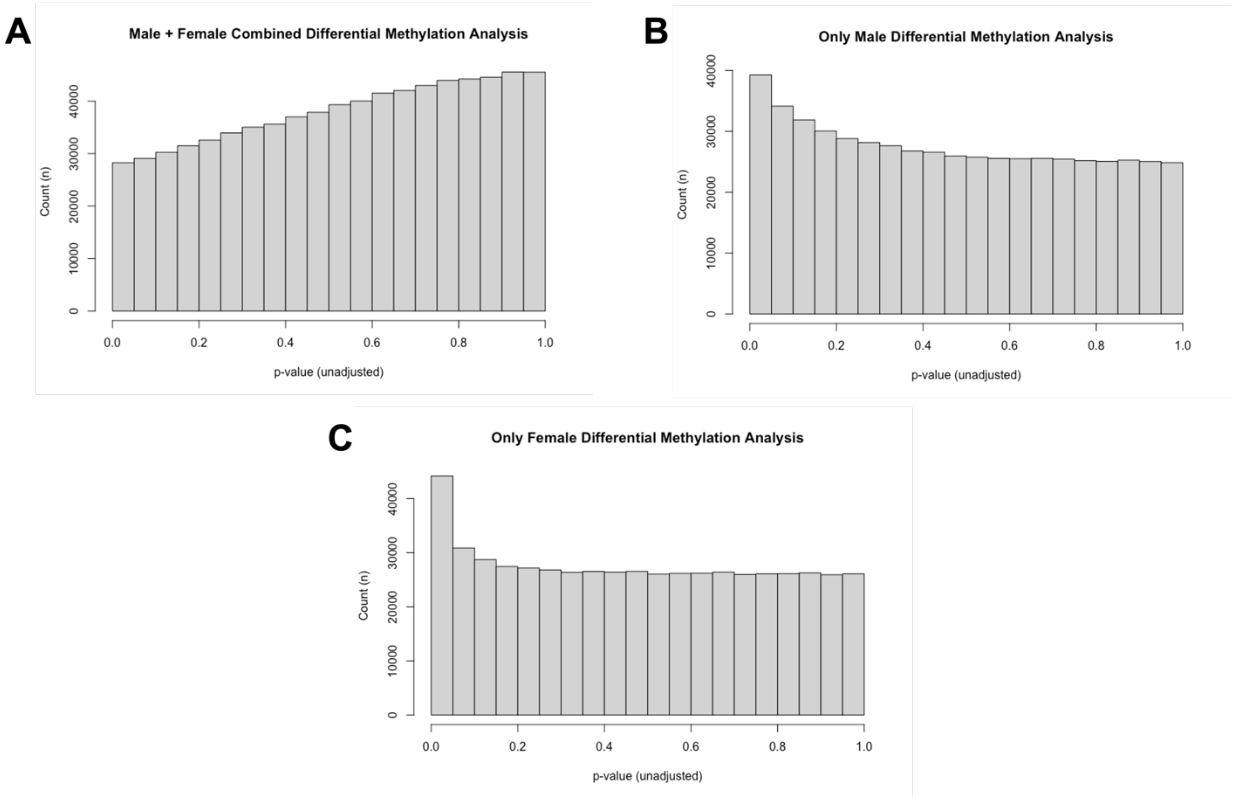

Figure S1: P-value histograms for hypothesis testing in combined and sex-stratified analyses. P-value histograms can be used as a quality control measure in association studies with a large number of hypothesis tests. The male+female hypothesis test histogram (A) shows a lack of uniformity, as well as skew towards high p-values, suggesting that our combined male + female modeling was biased and not accurately testing the hypothesis of differential DNA methylation by disease status. After stratifying by sex, the male (B) and female (C) p-value histograms are largely uniform, with a peak near zero, as would be expected from a well-calibrated hypothesis test. One way to account for differences in directionality by sex during modeling would be to include an interaction between sex and PD status, but that requires additional degrees of freedom which our modest sample size does not afford, and could lead to issues with modeling interpretation. As such, to remove the requirement for an interaction term and preserve statistical power, we elected to stratify our analyses by sex.
